# Deletion of *IKK2* in haematopoietic cells of adult mice leads to elevated interleukin-6, neutrophilia and fatal gastrointestinal inflammation

**DOI:** 10.1038/s41419-020-03298-9

**Published:** 2021-01-04

**Authors:** Karla C. Fischer, Carmel P. Daunt, Cédric S. Tremblay, Sheila Dias, James E. Vince, Anissa M. Jabbour

**Affiliations:** 1 The Walter and Eliza Hall Institute of Medical Research, Parkville, VIC Australia; 2 Department of Medical Biology, University of Melbourne, Parkville, VIC Australia; 3 Australian Centre for Blood Diseases, Monash University, Melbourne, VIC Australia

**Keywords:** Growth factor signalling, Interleukins, Myelopoiesis, Inflammatory diseases, Haematopoietic stem cells

## Abstract

The IκB kinase complex, consisting of IKK1, IKK2 and the regulatory subunit NEMO, is required for NF-κB signalling following the activation of several cell surface receptors, such as members of the Tumour Necrosis Factor Receptor superfamily and the Interleukin-1 Receptor. This is critical for haematopoietic cell proliferation, differentiation, survival and immune responses. To determine the role of IKK in the regulation of haematopoiesis, we used the Rosa26^Cre-ERT2^ Cre/lox recombination system to achieve targeted, haematopoietic cell-restricted deletion of the genes for IKK1 or IKK2 in vivo. We found that the IKK complex plays a critical role in haematopoietic cell development and function. Deletion of IKK2, but not loss of IKK1, in haematopoietic cells led to an expansion of CD11b/Gr-1-positive myeloid cells (neutrophilia), severe anaemia and thrombocytosis, with reduced numbers of long-term haematopoietic stem cells (LT-HSCs), short-term haematopoietic stem cells (ST-HSCs) and multipotential progenitor cells (MPPs), increased circulating interleukin-6 (IL-6) and severe gastrointestinal inflammation. These findings identify distinct functions for the two IKK catalytic subunits, IKK1 and IKK2, in the haematopoietic system.

## Introduction

Cytokine receptor signalling is essential for the survival, proliferation and differentiation of haematopoietic stem cells (HSCs) and their progeny. Cytokines control the development of haematopoietic progenitors into cells of the myeloid, lymphoid and erythroid lineages by stimulating cell proliferation and differentiation, as well as by inhibiting apoptotic cell death^1–3^. Binding of a cytokine to its receptor leads to the activation of multiple kinase signalling pathways, including the JAK/STAT, RAS–MAP kinase (MAPK) and PI3-kinase/AKT pathways^3–6^. The IκB kinase (IKK) complex, which consists of two serine/threonine kinases, IKK1 and IKK2, and the regulatory subunit NEMO^7–14^, plays an important role in cellular signal transduction networks as a signalling hub and interface for crosstalk between NF-κB activating pathways (canonical as well as non-canonical)^10,15^ and other processes that control cell proliferation, differentiation and survival^16,17^.

The signalling pathways leading to activation of the IKK complex following ligation of the members of the Tumour Necrosis Factor Receptor (TNFR) superfamily are well established^18^. For example, lymphotoxin-β (LT-β)^19^, B-cell activating factor (BAFF) and CD40L^20–22^ trigger activation of IKK1 and non-canonical NF-κB signalling. Conversely, inflammatory cytokines, such as tumour necrosis factor (TNF) and interleukin-1β (IL-1β), as well as certain mitogens or bacterial lipopolysaccharide activate IKK2 and canonical NF-κB signalling^23^.

Studies using gene-targeted mice revealed that IKK1 and IKK2 have distinct roles. IKK1 is required for NF-κB2 (p100) activation, B-cell maturation and the formation of secondary lymphoid organs^24,25^. Conversely, IKK2 is needed for canonical NF-κB signalling that promotes the survival of cells exposed to TNF or IL-1β^26,27^. Deletion of the regulatory subunit NEMO results in a phenotype similar to that seen in IKK2-deficient mice, characterised by failure to activate canonical NF-κB signalling, aberrant hepatocyte apoptosis and TNF hypersensitivity^28–30^.

Conditional, lineage-specific deletion of IKK2 in B cells (using CD19-Cre) led to defects in the maintenance of mature B lymphocytes, resulting in a significant reduction of all peripheral B-cell subsets^31,32^. T-cell lineage-specific deletion of IKK2 (using CD4-Cre or Lck-Cre), although not affecting naive T-cell development, impaired the generation of regulatory and memory T cells, with mature T cells being dependent on IKK-induced NF-kB activation for survival^33,34^. Deletion of IKK2 in haematopoietic (stem) cells using the interferon-inducible Mx1-Cre system resulted in an expansion of the myeloid cell compartment caused by a skewed lineage commitment, promoting myeloid over erythroid cell fate^35^. Moreover, IKK2 ablation in macrophages (using Mx1-Cre) diminished the survival of TLR4-activated macrophages^36,37^ and increased the severity of atherosclerosis in LDL receptor-deficient mice (using LysM-Cre)^38^, due to reduced IKK2-mediated NF-kB pathway activation.

In this study, we have sought to define the relative roles of IKK1 and IKK2 in haematopoietic cell development in vivo by using inducible deletion of either IKK1 or IKK2 in the haematopoietic system of adult mice.

## Results

### Deletion of *IKK1* or *IKK2* in the haematopoietic system of adult mice

**Fig. 1:**
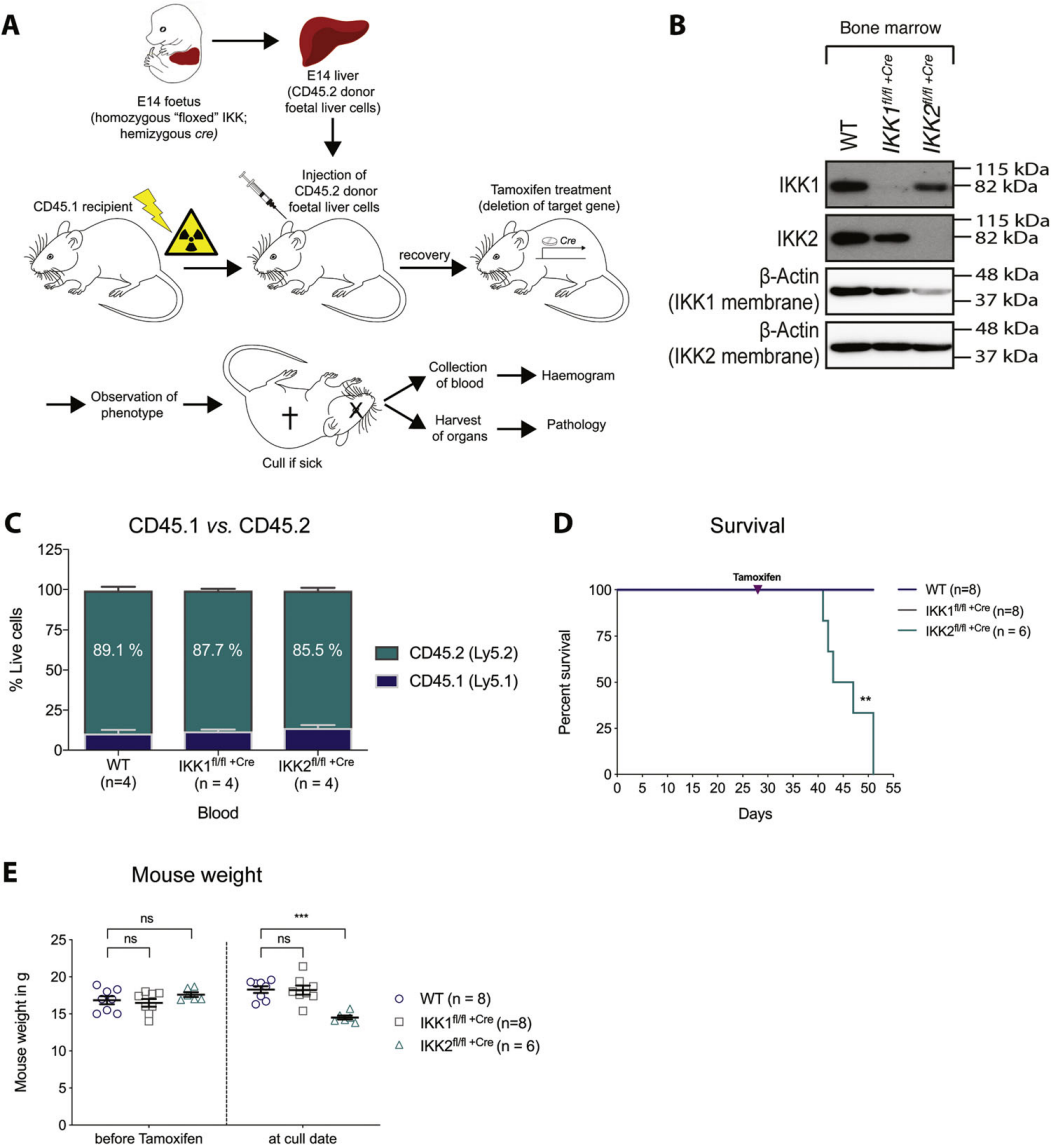
Deletion of IKK2 in haematopoietic stem cells in vivo causes lethality with mice showing significant weight loss. **A** Experimental design of the haematopoietic reconstitution model. E14 WT(IKK), IKK1^fl/fl^ or IKK2^fl/fl^ donor foetal liver cells hemizygous for ROSA26 Cre-ERT2 (CD45.2^+^) were injected into lethally irradiated WT recipient mice (CD45.1^+^). Reconstitution of peripheral blood cells by donor foetal liver cells was assessed by CD45.1/CD45.2 staining on day 28 post transplantation. The floxed target gene was deleted 4 weeks post transplant in vivo by oral gavage of tamoxifen (200 mg/kg) on two consecutive days. Mice were monitored for any signs of illness, such as weight loss and lethargy, and euthanised according to our animal ethics regulations. Blood and organs were collected at the experiential endpoint for analysis. **B** Western blot showing deletion of IKK1 or IKK2 in bone marrow from tamoxifen-treated mice at the experimental endpoint. Membranes were probed for IKK1 and IKK2. Probing for β-actin was used as a loading control. **C** Contribution of the transplanted donor (CD45.2^+^) foetal liver-derived cells to peripheral blood cells in recipient mice (mean + SEM, *n* = 4 for each genotype). Data are from one of three independent experiments that each tested 4 mice transplanted with foetal liver cells of each genotype. The graph is representative of a typical experiment. **D** Kaplan–Meier curve of WT, IKK1-deleted (IKK1^fl/fl +Cre^) and IKK2-deleted (IKK2^fl/fl +Cre^) mice post bone marrow transplantation. Curves of WT and IKK1-deleted mice exactly overlap. Data represent two independent experiments with *n* = 8 mice for WT and IKK1 knockout and *n* = 6 mice for IKK2 knockout. **E** Comparison of mouse body weight before tamoxifen administration (i.e. before gene deletion) and at the date of sacrifice. Values for individual mice are shown and data are expressed as means ± SEM of two independent experiments. ^ns^*p* > 0.05; **p ≤ 0.01 and ***p ≤ 0.001 as determined by **D** Mantel–Cox test (*p* = 0.0026) and **E** one-way ANOVA and Dunnett’s multiple comparison test (*p* < 0.0001).

To identify the roles of IKK1 and IKK2 in haematopoietic cell development, we transplanted murine E14 wild-type, IKK1-floxed ROSA26 Cre-ERT2^+^ or IKK2-floxed ROSA26 Cre-ERT2^+^ CD45.2^+^ foetal liver cells (a rich source of haematopoietic stem/progenitor cells (HSPCs)) into lethally irradiated wild-type CD45.1^+^ recipient mice (Fig. 1A). This model employs the Rosa26^Cre-ERT2^ Cre/lox recombination system to achieve targeted deletion of the gene of interest in haematopoietic cells (derived from the transplanted HSPCs) in vivo upon tamoxifen treatment. Four weeks after transplantation, successful reconstitution of the peripheral blood of recipients by donor foetal liver-derived cells was assessed by flow cytometry (CD45.1^+^ vs. CD45.2^+^) (Fig. 1C). In two independent cohorts each of 11 mice, an average reconstitution efficiency of at least 85% donor-derived cells was observed for all genotypes of donor cells. The floxed target gene was then deleted selectively in haematopoietic cells of the transplanted mice by oral administration of two doses of tamoxifen (200 mg/kg). IKK1 or IKK2 gene deletion in cells of the bone marrow was confirmed by Western blot analysis (Fig. 1B).

### Loss of IKK2 in haematopoietic cells caused significant weight loss and severe morbidity

To determine the effect of IKK1 or IKK2 deletion in the haematopoietic compartment, we monitored reconstituted mice (HSC WT^+Cre^, HSC *IKK1*^fl/fl +Cre^ and *IKK2*^fl/fl +Cre^, hereafter referred to as WT, IKK1^fl/fl +Cre^ or IKK2^fl/fl +Cre^, respectively) for 23 days after tamoxifen-induced gene deletion. Across two independent cohorts, induced deletion of IKK2, but not loss of IKK1, in haematopoietic cells resulted in ≥20% weight loss, the predetermined ethical endpoint, within approximately 1–3 weeks after tamoxifen treatment (Fig. 1D, E). None of the mice in which the IKK2 gene had been deleted in haematopoietic cells recovered, with the last mouse needing to be euthanised for ethical reasons 23 days after tamoxifen treatment. In contrast, mice with a WT or an IKK1-deleted haematopoietic system did not show weight loss or any sign of disease, with their body weight gradually increasing over the course of the experiment.

### Deletion of *IKK2* in the adult mouse haematopoietic system results in thrombocytosis and normocytic anaemia

**Fig. 2:**
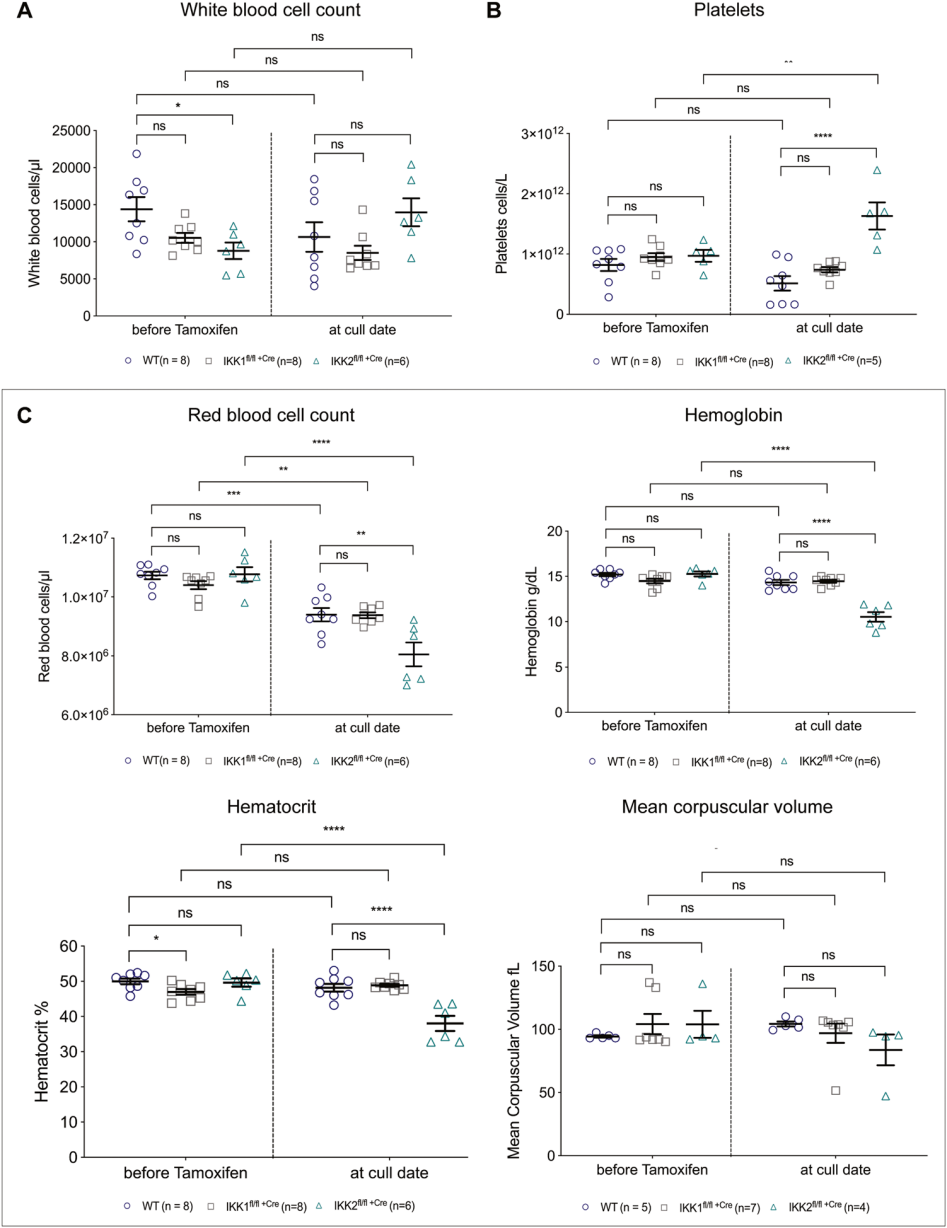
IKK2^fl/fl +Cre^ mice have a normal white blood cell count but develop normocytic anaemia, presenting with significantly reduced red blood cell counts, lower haemoglobin levels and a decreased haematocrit, whereas the mean corpuscular volume (MCV) is in the normal range. IKK2^fl/fl +Cre^ mice further but present with thrombocytosis, evident by elevated platelet counts. **A** White blood cell count (WBC), **B** platelet count (PLT) and **C** red blood cell parameters, including total red blood cell count (RBC), haemoglobin concentration (HGB), haematocrit (HCT) and mean corpuscular volume (MCV), as assessed on the Avida 2120 haematological analyser. Individual values, mean and SEM are shown of two independent experiments. ^ns^*p* > 0.05, **p ≤ 0.01 and ****p ≤ 0.0001 as determined by one-way ANOVA (WT^Cre+^ at sacrifice vs. IKK2^fl/fl Cre+^ at sacrifice: WBC *p* = 0.1024; PLT *p* < 0.0001; RBC *p* = 0.0019; HGB *p* = <0.0001; HCT *p* < 0.0001; MCV *p* = 0.2707) and Dunnett’s multiple comparison test.

At the experimental endpoint, the mice of the different genotypes had similar total white blood cell counts (Fig. 2A) but those whose haematopoietic cells had been deleted for *IKK2* had a 3-fold increase in platelet numbers compared to WT mice (Fig. 2B). Moreover, as the *IKK2*-deleted mice lost weight, they started to display signs of anaemia, such as pale paws. At the experimental endpoint, *IKK2*-deleted mice had a 15% lower red blood cell count, 25% lower haemoglobin levels and a 20% lower haematocrit compared to control animals (Fig. 2C). The mean corpuscular volume was within the normal range, indicating normocytic anaemia. Consistent with the reduced red blood cell counts, the bone marrow from *IKK2*-deleted mice was very pale compared to that of *IKK1-* deleted mice. In comparison, the red blood cell parameters were found to be within the normal range for the WT and *IKK1*-deleted mice, despite minor tamoxifen-induced toxicity on erythropoiesis that resulted in a slightly reduced RBC count early after drug administration. This minor toxic effect on erythropoiesis was transient and has been previously reported for mice on a C57BL/6 background and would have affected all mice within the experiment to a similar extent independent of their genotype^39^.

### Mice lacking *IKK2* in their haematopoietic cells develop neutrophilia and monocytosis

**Fig. 3:**
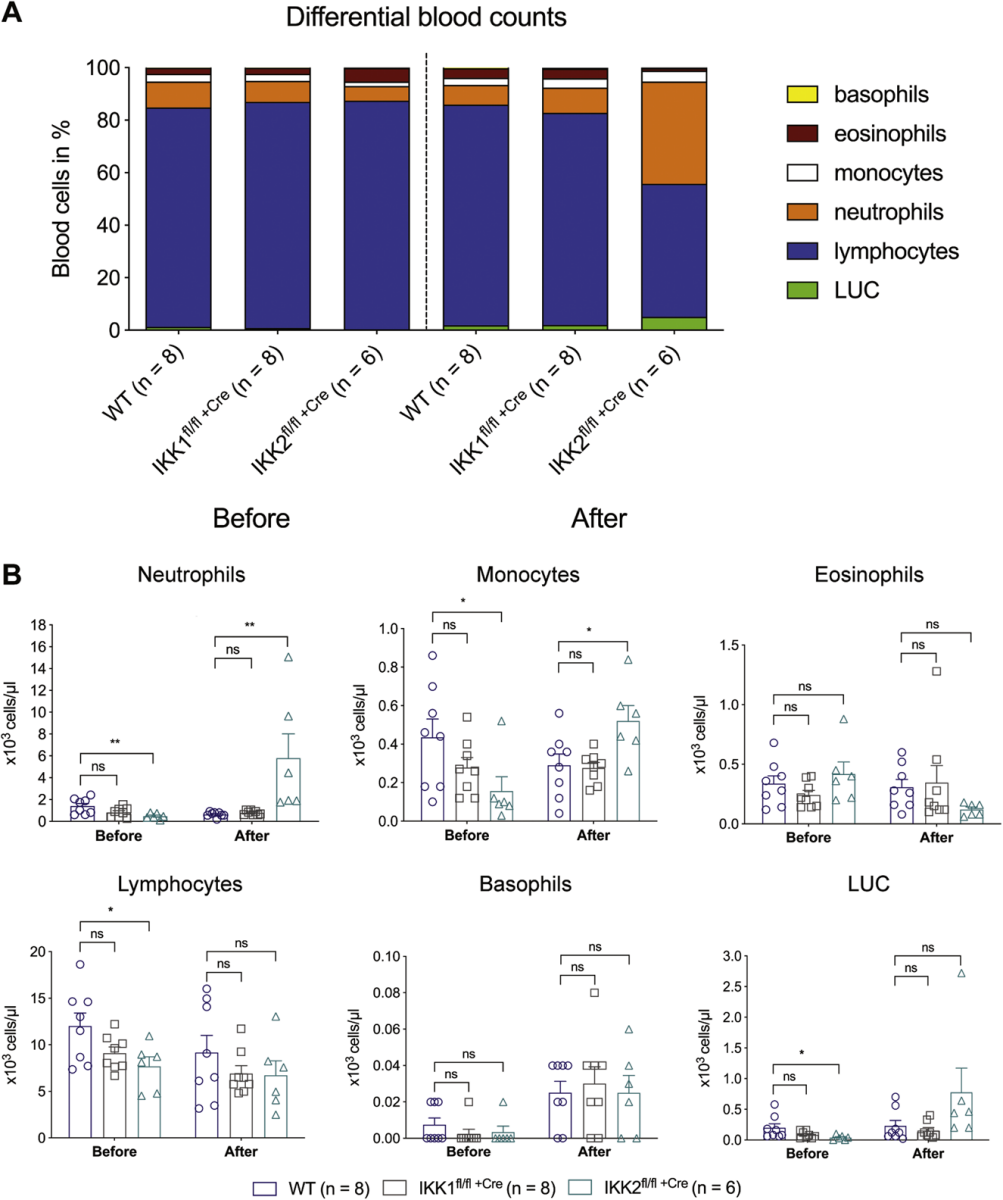
Loss of IKK2 in haematopoietic stem cells results in neutrophilia, monocytosis and eosinopenia in peripheral blood. Analysis of peripheral blood profile in control and IKK-floxed mice before and after gene deletion in haematopoietic cells at the time of sacrifice. **A** Differential white blood cell counts, **B** total white blood cell counts. Neutrophil, monocyte, eosinophil, lymphocyte, basophil and large peroxidase-negative cell (LUC) count as assessed on the Avida 2120 haematological analyser. Data are presented as means (**A**) and means + SEM (**B**) from 6 to 8 mice of each genotype of two independent experiments. Graphs are representative of a typical experiment. ^ns^*p* > 0.05; *p ≤ 0.05 and **p ≤ 0.01 as determined by one-way ANOVA (WT^Cre+^ after tamoxifen treatment vs. IKK2^fl/fl Cre+^ after tamoxifen treatment: neutrophils *p* = 0.0048; monocytes *p* = 0.0141; eosinophils *p* = 0.2922; lymphocytes *p* = 0.4282; basophils *p* = 0.8841; LUC *p* = 0.0896) and Dunnett’s multiple comparison test.

Induced deletion of *IKK2* in haematopoietic cells caused neutrophilia and monocytosis (Fig. 3A), with the IKK2-deleted mice having on average a 7-fold increase in absolute neutrophil numbers and a 2-fold increase in absolute monocyte numbers compared to the IKK1-deleted mice and a 9-fold increase in absolute neutrophil numbers and a 1.5-fold increase in absolute monocyte numbers compared to the WT mice, respectively (Fig. 3B). Some IKK2^fl/fl +Cre^ mice also displayed an elevated number of large unstained cells that were likely to be activated lymphocytes (Fig. 3B). In contrast, the WT and IKK1^fl/fl +Cre^ mice presented with normal blood cell parameters.

### Induced IKK2 deletion in haematopoietic cells caused B-cell lymphocytopenia, T-cell lymphocytosis, granulocytosis, erythrocytopenia and natural killer cell deficiency

**Fig. 4:**
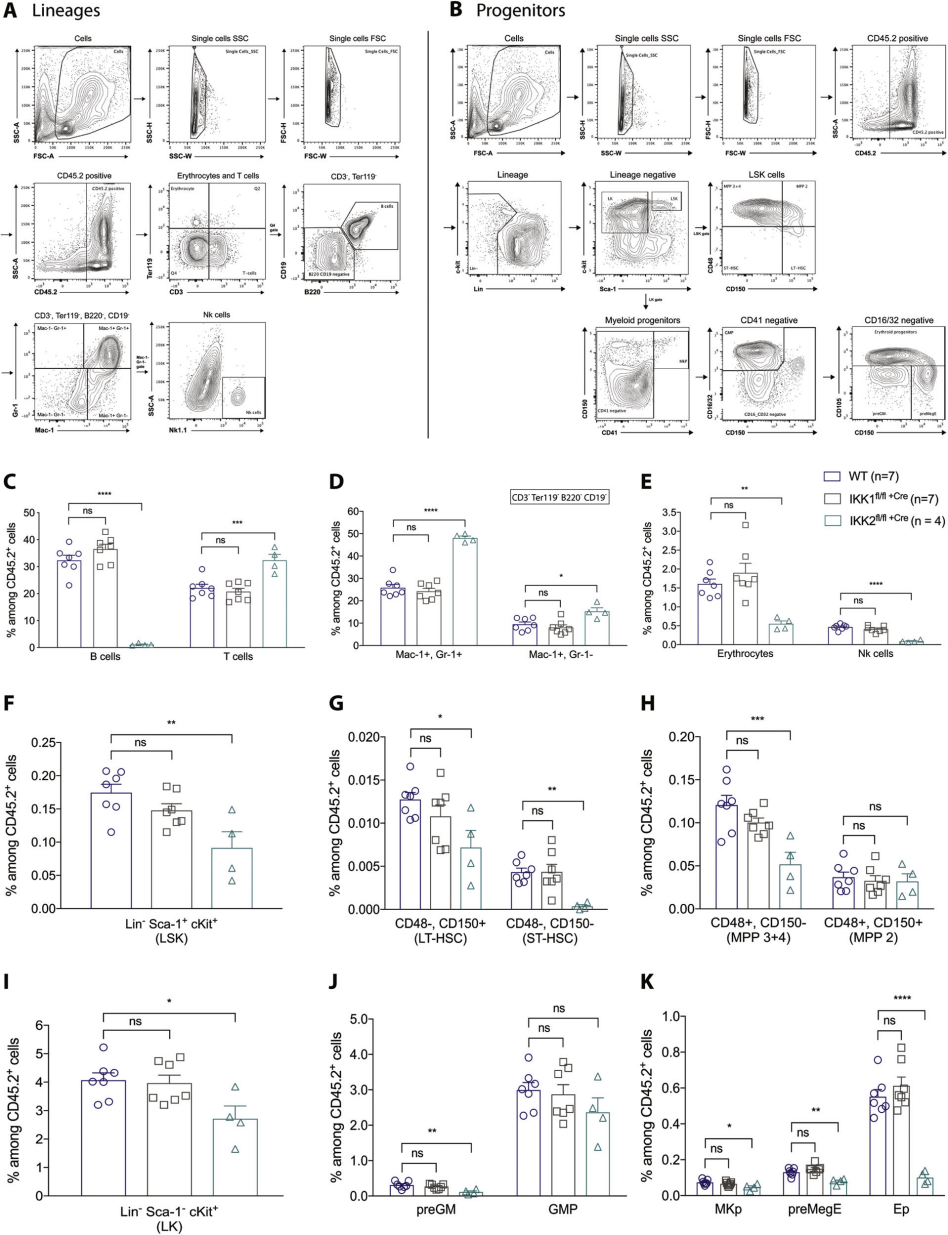
IKK2 deletion in haematopoietic stem cells results in B-cell lymphocytopenia, T-cell lymphocytosis, granulocytosis, erythrocytopenia and natural killer cell deficiency. Moreover, IKK2-deleted mice exhibit reduced numbers of long- and short-term haematopoietic stem cells (HSCs), multipotential progenitors (MPPs), pre-granulocyte–macrophage progenitors, megakaryocyte progenitors (MKp), pre-megakaryocyte–erythroid progenitors (preMegE) and erythroid progenitors (Ep) in the bone marrow. **A**, **B** Representative flow cytometric plots to examine the haematopoietic compartment of mice of the indicated genotypes. **C**–**K** Flow cytometric analysis of total bone marrow harvested from control and IKK1- or IKK2-deleted mice, respectively, at the experimental endpoint. Percentages of **A** B and T cells, **B** myeloid populations Mac-1^+^/Gr-1^+^ and Mac-1^+^/Gr-1^−^, **C** nucleated erythrocytes and natural killer (Nk) cells, **D** Lin^−^ Sca-1^+^ c-Kit^+^ (LSK) cells, **E** CD48^−^ CD150^+^ long-term (LT) HSCs and CD48^−^ CD150^−^ short-term (ST) HSCs, **F** CD48^+^ CD150^−^ MPP3 +4 and CD48^+^ CD150^−^ MPP2 cells, **G** Lin^−^ Sca-1^−^ c-Kit^+^ (LK) cells, **H** pre-granulocyte–macrophage progenitors (pre-GM) and granulocyte–macrophage progenitors (GMP) and **I** megakaryocyte progenitors (MKp) and pre-megakaryocyte–erythroid progenitors (preMegE) and erythroid progenitors (Ep) among CD45.2^+^ cells in the bone marrow. The results are presented as means ± SEM from 4 to 7 mice of each genotype of two independent experiments. Graphs are representative of a typical experiment. ^ns^*p* > 0.05, *p ≤ 0.05, **p ≤ 0.01, ***p ≤ 0.001 and ****p ≤ 0.0001 as determined by one-way ANOVA (B cells *p* < 0.0001; T cells *p* = 0.0004; Mac-1^+^, Gr-1^+^ *p* < 0.0001; Mac-1^+^, Gr^-^1^−^ *p* = 0.0045; erythrocytes *p* = 0^.^0015; Nk cells *p* < 0.0001; LSK *p* = 0.0061; LT-HSC *p* = 0.0400; ST-HSC *p* = 0.0031; MPP3 +4 *p* = 0.0016; MPP2 *p* = 0.8501; LK *p* = 0.0258; pre-GM *p* = 0.0041; GMP *p* = 0.3527; MKp *p* = 0.0203; preMegE *p* = 0.0003; Ep *p* < 0.0001) and Dunnett’s multiple comparison test.

To determine whether the changes in peripheral blood had resulted from changes in the bone marrow, cells from this tissue harvested at the experimental endpoint were stained with antibodies against lineage-specific cell surface markers (Fig. 4A, B, Supplementary Fig. S1). Absolute numbers of B cells in the marrow of IKK2 -deleted mice were found to be 28-fold decreased compared to control mice (Fig. 4C). This is consistent with previous reports that B-lineage-specific deletion of IKK2 causes B-cell lymphocytopenia due to defects in the maintenance of mature B cells^31^. Our IKK2-deleted mice also had only a fifth of the number of natural killer (NK) cells in the bone marrow compared to WT mice (Fig. 4E). In contrast, they had 45% more T cells than control mice and 55% more T cells than IKK1^fl/fl +Cre^ mice at the same time after tamoxifen treatment (Fig. 4C). Significant expansions of both the Mac-1^+^ Gr-1^+^ and Mac-1^+^ Gr-1^–^ myeloid cell populations were observed in the bone marrow of IKK2-deleted mice. This confirms the findings from the peripheral blood tests and suggests that IKK2 deletion in haematopoietic cells results in granulocytosis (Fig. 4D). This finding is consistent with previous reports showing that deletion of IKK2 in the haematopoietic compartment causes myeloid cell expansions^35^. Consistent with the anaemia observed in the peripheral blood of IKK2-deleted mice, a significant reduction in the proportion of Ter-119^+^-nucleated erythroid cells was observed in the bone marrow of these animals (Fig. 4E).

### *IKK2*-deleted mice have reduced numbers of long- and short-term haematopoietic stem cells (HSCs) and fewer multipotential progenitors (MPPs) in the bone marrow

To examine the impact of the deletion of select IKK subunits on the HSPC compartment, we compared the bone marrow between wild-type control, IKK1^fl/fl +Cre^ and IKK2 ^fl/fl +Cre^ mice with respect to the abundance of Lin^−^ Sca-1^+^ c-Kit^+^ (LSK), Lin^−^ c-Kit^+^ (LK), multipotential progenitor (MPP), granulocyte/monocyte progenitor and megakaryocyte/erythroid progenitor (MEP) cells.

Deletion of IKK2, but not deletion of IKK1, resulted in a 50% reduction of the numbers of LSK and long-term HSC (LT-HSC) cells, a 12-fold reduction in the numbers of short-term (ST-HSC) and a 50% reduction of the numbers of MPP3 +4 cells, but there was no significant difference in the numbers in the MPP2 cell population (Fig. 4F–H). Surprisingly, the results from our analysis of IKK2 ^fl/fl +Cre^ mice are opposite to those previously reported by Zhang et al. using the Mx1-Cre model applying poly(I:C) treatment to achieve gene deletion in HSCs. They reported increased numbers of LSK, LT- and ST-HSC and MPP cells upon IKK2 deletion^35^.

### Mice deleted for IKK2 in their haematopoietic cells exhibit reduced numbers of pre-granulocyte–macrophage progenitors, megakaryocyte progenitors (MKp), pre-megakaryocyte–erythroid progenitors (preMegE) and erythroid progenitors (Ep) in the bone marrow

Contrary to Zhang et al.^35^, we found that IKK2-deleted mice had a 1.5-fold decrease in the numbers of LK cells, a 3-fold decrease in the numbers of pre-granulocyte–macrophage progenitors (pre-GM), a 1.5-fold reduction in the numbers of megakaryocyte progenitors (MKp), a 1.5-fold reduction in the numbers of pre-megakaryocyte–erythroid progenitors (preMegE) and 5-fold less erythroid progenitors (Ep) in comparison to control and the IKK1-deleted mice (Fig. 4I–K). With the exception of the results obtained for the erythroid progenitors, the findings for the other cell populations that were examined are the opposite of those published by Zhang et al.^35^.

### Mice deleted for IKK2 in their haematopoietic cells present with splenomegaly, infiltration of haematopoietic cells into tissues and severe inflammation of the gastrointestinal tract causing lethality

**Fig. 5:**
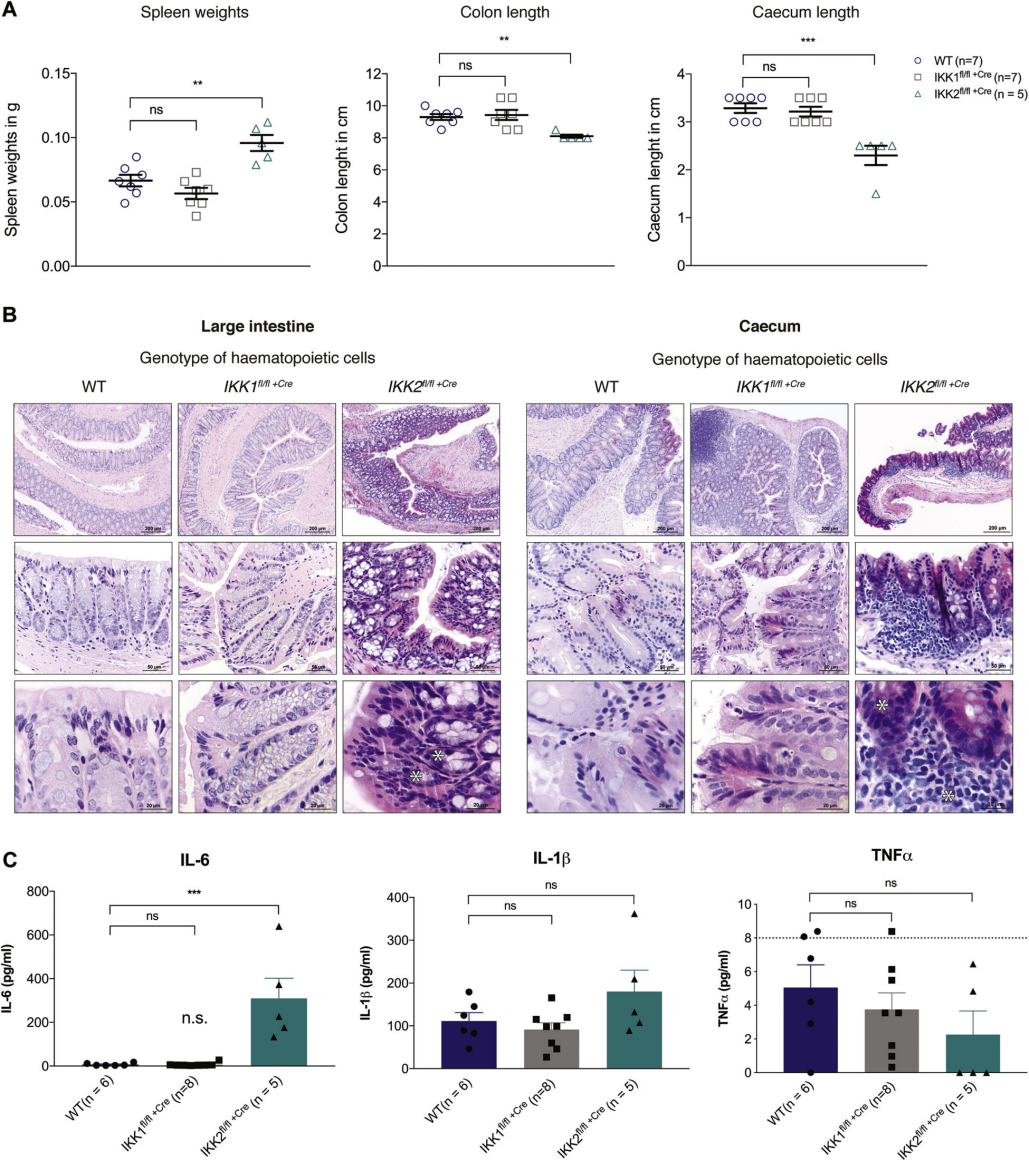
Deletion of IKK2 in haematopoietic cells results in severe inflammation of the gastrointestinal tract and elevated serum levels of the pro-inflammatory cytokine interleukin-6. **A** Spleen weight, colon length and caecum length were measured in mice of the indicated genotypes at the experimental endpoint. Individual values, mean and SEM are shown of two independent experiments, with *n* = 7 mice for control and IKK1-deleted, and *n* = 5 mice for IKK2-deleted mice. **B** Colon tissue or caecum tissue, respectively, was harvested from control, IKK1- and IKK2-deleted mice at the experimental endpoint, fixed in 10% buffered formalin, sectioned and stained with haematoxylin and eosin. Images were acquired with a microscope system at 10×, 40× and 100× (oil immersion) and evaluated microscopically. Images are of three representative mice of each genotype. Asterisks indicate infiltrating polymorphonuclear leucocytes present in the colon or caecum tissue of IKK2-deleted mice. **C** ELISA analysis of the concentrations of IL-6, IL-1β and TNFα in the sera of mice of the indicated genotypes at the experimental endpoint. The dotted line indicates ELISA detection limit. Data are shown as mean ± SEM of two independent experiments, including individual values with *n* = 6 mice for control mice, *n* = 8 mice for IKK1 -deleted mice and *n* = 5 mice for IKK2-deleted mice. ^ns^*p* > 0.05, **p ≤ 0.01 and ***p ≤ 0.001 as determined by one-way ANOVA (Spleen *p* = 0.0002; Colon *p* = 0.0038; Ceacum *p* = 0.0001; IL-6 *p* = 0.0002; IL-1β *p* = 0.1006; TNFα *p* = 0.3400) and Dunnett’s multiple comparison test.

To determine the cause of morbidity in the IKK2-deleted mice, organs were harvested from control, IKK1- and IKK2-deleted mice at the experimental endpoint and evaluated histopathologically. The IKK2-deleted mice had a 1.5-fold increase in spleen weights compared to control and IKK1-deleted mice (Fig. 5A). IKK1^fl/fl +Cre^ and IKK2^fl/fl +Cre^ mice had similar sized livers with liver weights within the normal range (data not shown). The large colons and caeca observed in mice in which IKK2 had been deleted in the haematopoietic cells were consistently shorter than those from mice in which IKK1 was deleted, which were similar to those seen in mice reconstituted with WT bone marrow cells (Fig. 5A). On average, mice whose haematopoietic cells had been deleted for IKK2 had 13% shorter colons and 30% shorter caeca compared to WT and IKK1-deleted mice, and these tissues appeared red and inflamed. Reductions in colon length in conjunction with red colour are signs of gut inflammation. Accordingly, analysis of haematoxylin and eosin (H&E)-stained colon and caecum sections (Fig. 5B) confirmed that mice whose HSCs have been deleted for IKK2 suffered from severe gut inflammation, evident by infiltration with inflammatory polymorphonuclear leucocytes. In contrast, control mice and mice whose haematopoietic cells had been deleted for IKK1 did not show any signs of gut inflammation and remained healthy throughout the course of the experiment. These results suggest that mice whose haematopoietic cells had been deleted for IKK2 became sick as a result of severe inflammation in their gastrointestinal tract.

### Loss of IKK2 in the haematopoietic system results in elevated interleukin-6 serum levels

To determine whether aberrant increases in pro-inflammatory cytokines could be the underlying cause of the intestinal inflammation in mice whose haematopoietic cells had been deleted for IKK2, serum concentrations of interleukin-1 beta (IL-1β), IL-6 and TNFα were measured by enzyme-linked immunosorbent assay (ELISA) at the experimental endpoint. On average 310 pg/mL IL-6 was detected in the sera of IKK2-deleted mice, whereas in sera from control and IKK1-deleted mice, only 7 pg/mL of IL-6 were detected (Fig. 5C). In contrast, the serum concentrations of IL-1β and TNFα were found to be within the normal range in all animals, with no significant differences detected between mice of the three genotypes. Given previous studies implicating IL-6 in intestinal inflammation, these findings indicate that pathologically elevated levels of the pro-inflammatory cytokine IL-6 may contribute to the severe intestinal inflammation observed in the IKK2-deleted mice.

### The addition of wild-type HSCs prevents the disease caused by loss of IKK2 in haematopoietic cells in a competitive reconstitution setting

**Fig. 6:**
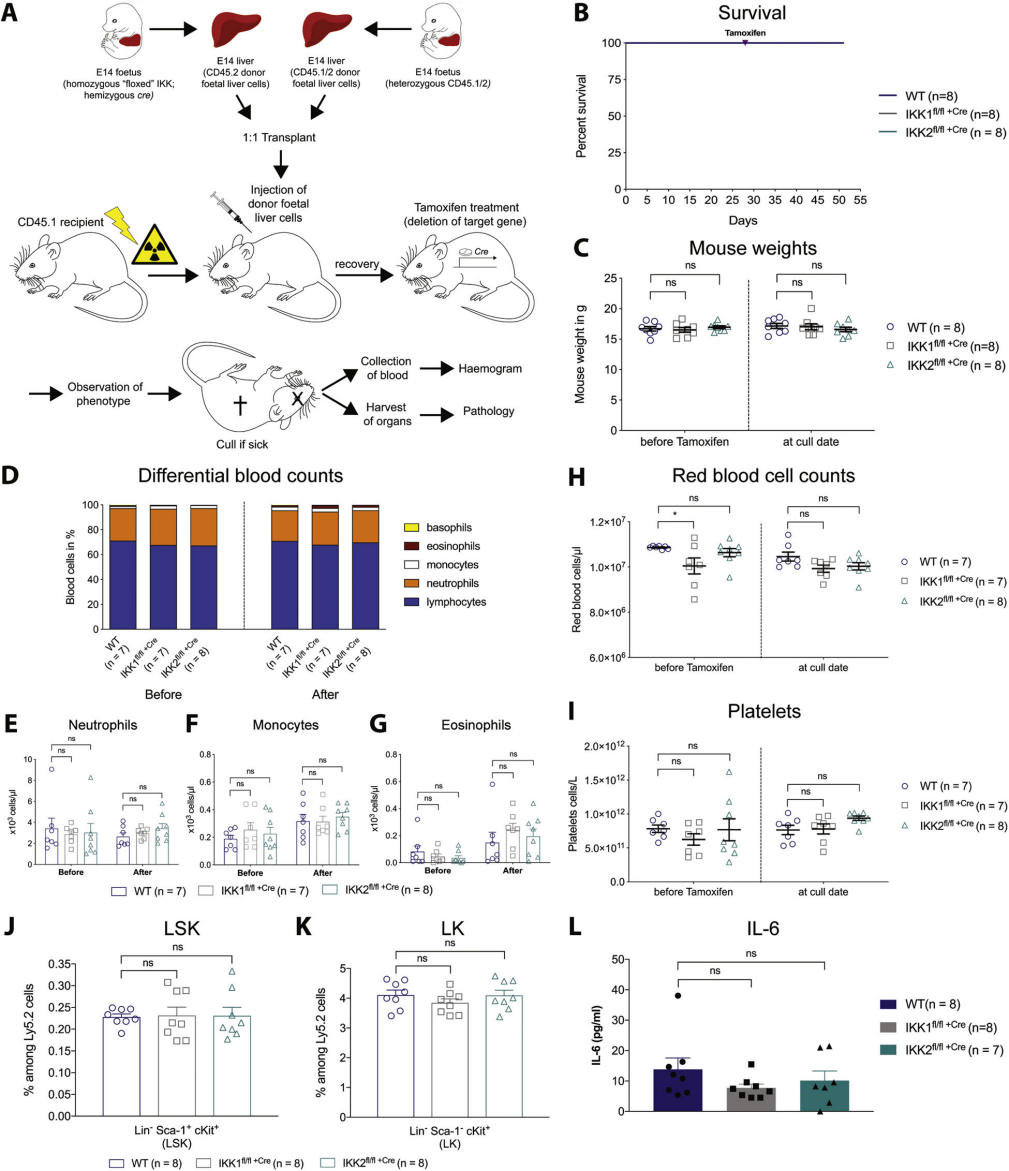
The presence of normal haematopoietic cells prevents the disease caused by deletion of IKK2 in haematopoietic cells. **A** Experimental design of competitive reconstitution assay. E14 WT(IKK), IKK1^fl/fl^ or IKK2^fl/fl^ donor 1 foetal liver cells hemizygous for ROSA26 Cre-ERT2 (CD45.2^+^) were mixed with E14 donor 2 WT (CD45.1^+^/CD45.2^+^) foetal liver cells at a 1:1 ratio, and co-transplanted into lethally irradiated WT (CD45.1+) recipient mice. Reconstitution of peripheral blood cells by the donor foetal liver cells was assessed by CD45.1/CD45.2 staining on day 28 post transplantation. Four weeks after transplantation, the mice were treated with tamoxifen via oral gavage on two consecutive days to delete the floxed target gene in 50% of the haematopoietic cells. Mice were then monitored for any signs of illness, such as weight loss and lethargy. Three weeks after initiation of treatment, mice were sacrificed, and blood and organs were collected for analysis. Peripheral blood and bone marrow were subject to flow cytometric analysis using the indicated surface markers. Organs were fixed in 10% buffered formalin, sectioned and stained with haematoxylin and eosin and assessed microscopically. **B** Kaplan–Meier curve of mice of the indicated genotypes. **C** Comparison of mouse body weight before tamoxifen administration (gene deletion) and at the date of sacrifice. Values of individual mice are shown and data are expressed as means ± SEM of one independent experiment. **D** Differential white blood cell counts, **E**–**G** total white blood cell counts. **E** Neutrophil, **F** monocyte and **G** eosinophil count as assessed on the Hemavet blood analyser. Data are presented as means (D) and means ± SEM (E–G) from 8 mice of each genotype of one independent experiment. **H** Peripheral red blood cell count (RBC) and **I** platelet cell count (PLT). Individual values, mean and SEM are shown of one independent experiment with *n* = 8 mice for each genotype. **J**, **K** Flow cytometric analysis of total bone marrow harvested from mice of the indicated genotypes at the experimental endpoint. Percentages of **J** Lin^−^ Sca-1^+^ c-Kit^+^ (LSK) cells and (K) Lin^−^ Sca-1^−^ c-Kit^+^ (LK) cells among CD45.2^+^ cells in the bone marrow. The results are presented as means ± SEM from eight mice of each genotype of one independent experiment. **L** ELISA analysis of serum levels of IL-6 in mice of the indicated genotypes at the experimental endpoint. Data are shown as mean ± SEM of one independent experiment, including individual values with *n* = 8 mice for control and IKK1 deleted in 50% of the haematopoietic cells and *n* = 7 mice for IKK2 deleted in 50% of the haematopoietic cells. ^ns^*p* > 0.05 as determined by one-way ANOVA (mouse weight *p* = 0.5956; neutrophils *p* = 0.3413; monocytes *p* = 0.7830; eosinophils *p* = 0.5920; RBC *p* = 0.1025; PLT *p* = 0.0940; LSK *p* = 0.9876; LK *p* = 0.4309; IL-6 *p* = 0.3313) and Dunnett’s multiple comparison test.

To test whether the presence of normal haematopoietic cells could prevent the abnormalities and pathology caused by the loss of IKK2 in haematopoietic cells, we performed competitive haematopoietic reconstitution experiments. Murine E14 wild-type, IKK1-floxed ROSA26 Cre-ERT2^+^ or IKK2-floxed ROSA26 Cre-ERT2^+^ CD45.2^+^ foetal liver cells that carried the ROSA26 Cre-ERT2 transgene were mixed at a 1:1 ratio with wild-type CD45.1/CD45.2 foetal liver cells and transplanted into lethally irradiated wild-type CD45.1^+^ recipient mice (Fig. 6A). Four weeks after transplantation, successful reconstitution of peripheral blood of recipients by donor foetal liver-derived cells was verified by flow cytometry (CD45.1^+^ vs. CD45.2^+^) (Supplementary Fig. S2). The floxed target gene was then deleted in vivo by oral administration of tamoxifen (200 mg/kg). Mice were euthanised at the experimental endpoint (weight loss ≥20% of starting body weight) according to the relevant animal ethics guidelines.

In contrast to IKK2 deletion in all haematopoietic cells, IKK2 deletion in only 50% of the haematopoietic cells in a competitive reconstitution setting did not result in detectable abnormalities and disease (Fig. 6B, Supplementary Fig. S3). All of these mice remained healthy throughout the course of the experiments, with no signs of weight loss (Fig. 6C). Mice deleted for IKK2 in 50% of their haematopoietic cells did not display any signs of neutrophilia (Fig. 6E), monocytosis (Fig. 6F), normocytic anaemia (Fig. 6H, Supplementary Fig. S4), thrombocytosis (Fig. 6I) or changes in key HSC populations (Fig. 6J, K, Supplementary Fig. S5). Moreover, no increase in the concentrations of IL-6 was detected in the sera of mice deleted for IKK2 in only 50% of their haematopoietic cells (Fig. 6L, Supplementary Fig. S6). These results show that loss of IKK2 in adult haematopoietic cells and the resulting disease, including intestinal inflammation and neutrophilia, can be prevented by the presence of wild-type haematopoietic cells.

## Discussion

Here, we show that IKK2 plays a crucial role in haematopoietic cell development, myeloid differentiation and blood cell maturation. In our study, we directly compared for the first time the functions of IKK1 and IKK2 in the haematopoietic system in vivo. Our findings demonstrate that IKK2 loss, but not loss of IKK1, in adult haematopoietic cells, results in severe neutrophilia, normocytic anaemia and thrombocytosis in peripheral blood, reflecting a skewed lineage commitment, promoting myeloid over erythroid cell fate. Deletion of IKK2 also led to an expansion of CD11b/Gr-1-positive myeloid cells (neutrophilia) and reduced numbers of LT-HSCs, ST-HSCs and MPP cells in the bone marrow, increased circulating interleukin-6 (IL-6) and severe gastrointestinal inflammation with weight loss, necessitating euthanasia.

Our results confirm previous reports of an expansion of the myeloid compartment caused by IKK2 deletion in adult mice, manifested in markedly increased circulating neutrophils in peripheral blood, impairment of erythropoiesis and thrombocytosis^35,40–42^. However, in contrast to previous reports stating that IKK2-deleted mice only display mild signs of anaemia and thrombocytosis^35,40^, our study revealed that animals whose haematopoietic cells had been deleted for IKK2 suffer from severe anaemia and thrombocytosis manifested in significantly decreased erythrocyte counts and significantly elevated platelet counts in comparison to control animals. Analysis of red blood cell parameters revealed that the anaemia can be classified as normocytic anaemia^43^, a type of anaemia that in most cases is caused by decreased production of normally sized red blood cells due to suppression of the production of erythrocytes. In our study, this was most likely provoked by a lack of erythrocyte progenitors in mice whose haematopoietic cells had been deleted for IKK2, due to a skewed lineage commitment. Flow cytometric analysis of the haematopoietic compartment revealed that the skewed lineage commitment seen in the peripheral blood of IKK2-deleted but not IKK1-deleted mice can also be observed in the bone marrow. IKK2-deleted mice presented with B-cell lymphocytopenia, T-cell lymphocytosis, natural killer cell deficiency, erythrocytopenia and granulocytosis. This identifies a unique role for the IKK2 catalytic subunit in haematopoietic cells. B-cell deficiency caused by a defect in the maintenance of mature B cells upon deletion of IKK2 in HSCs has been previously reported in the literature, as has the development of erythrocytopenia and granulocytosis upon IKK2 deletion in HSCs^31,35,42^. Interestingly, however, our results show additionally that loss of IKK2 in haematopoietic cells also results in T-cell lymphocytosis and natural killer (NK) cell deficiency, two features of IKK2-deficient haematopoiesis that have not yet been described in the literature. Of note, the increased percentages of CD3-positive T cells observed in the bone marrow of wild-type and IKK1-deleted mice (around 20%) were unexpected since the bone marrow of wild-type mice usually harbours fewer than 5% CD3-positive T cells. We speculate that this higher-than-usual percentage of T cells found in the bone marrow of these mice might be related to tamoxifen treatment.

Apart from a marked expansion of CD11b/Gr-1-positive myeloid cells, our study also shows that IKK2-deleted mice have reduced numbers of long- and short-term haematopoietic stem cells and fewer MPP. This suggests that loss of IKK2 results in a defect in the maintenance of stem cells, leading to a depletion of the stem cell pool over time, ultimately resulting in bone marrow failure. To further refine the analysis of the haematopoietic stem cell populations in these mice, additional markers, such as CD34 and FLT3, could be used in follow-up experiments. Histopathological analysis of tissues, such as spleen and liver, showed that IKK2-deleted mice, but not IKK1-deleted mice presented with clear signs of extramedullary haematopoiesis (as demonstrated by the presence of nucleated erythrocytes and megakaryocytes in these two tissues). Most likely, this represents an attempt to compensate for bone marrow failure, consistent with previous findings^42^. Further analysis of the MPP compartment revealed that compared to control animals, mice whose haematopoietic cells had been deleted for IKK2 had fewer MPP3 +4 progenitor cells, whereas their MPP2 counts were not significantly altered. All three MPP subsets are produced independently by HSCs at variable levels, depending on demand, to sustain blood cell production, with MPP2 and MPP3 being myeloid-biased subsets and MPP4 being a lymphoid-primed MPP subset^44^. Within the haematopoietic hierarchy, each MPP can be positioned upstream of their respective further differentiated progeny, with MPP2 upstream of pre-megakaryocyte/erythroid (Pre-Meg) and MPP3 upstream of pre-granulocyte/macrophage (Pre-GM) cells, respectively^44^. Since IKK2-deleted mice present with B-cell lymphocytopenia and natural killer cell deficiency, it is reasonable to assume that the decrease in the numbers of the MPP3 +4 cells of IKK2-deficient mice is a result of a significant loss of MPP4 progenitor cells due to the myeloid lineage skewing observed in IKK2-deleted mice. This loss of MPP4 progenitor cells would result in overall reduced numbers of this combined MPP3 +4 cell population. Moreover, since our analysis has shown no significant differences in the numbers of the MPP2 progenitor populations between mice of the different genotypes, it appears likely that the block in erythrocyte differentiation observed in the IKK2-deleted mice occurs at the MEP stage. This would skew lineage commitment toward megakaryocytopoiesis at the expense of erythropoiesis, resulting in the loss of erythroid progenitors and an increase in megakaryocyte progenitors. This would explain the erythropenia and thrombocytosis observed in the IKK2-deleted mice.

Our finding that IKK2 deletion in haematopoietic cells results in a loss of progenitor cells contradicts a previous study published by Zhang et al.^35^ where they reported an increase in ST-HSCs and MPP cell numbers in mice following IKK2 gene deletion. These contradictory results together with the differences seen in the severity of the observed anaemia and thrombocytosis in our work compared to the study published by Zhang et al.^35^ most likely are a result of the different methods used to achieve *Ikk2* gene deletion in vivo. Zhang et al.^35^ employed the *Myxovirus resistance-1 (Mx1-Cre)* system, where the Mx dynamin-like GTPase (*Mx1*) promotor is activated in an interferon-dependent manner following injection of polyinosinic:polycytidylic acid (poly I:C), a synthetic double-stranded RNA that mimics viral infection, resulting in the induced expression of the *Cre* recombinase transgene. Despite being a “deleter strain” that is commonly used in experimental haematology to achieve gene deletion in haematopoietic stem cells, this system is known to have caveats, such as activation of an interferon response^45^, which impacts HSCs independently of deletion of the floxed gene of interest. This may explain the differences between the findings from the Zhang et al. study^35^ and our results using tamoxifen-inducible activation of a latent Cre recombinase (RosaCreERT2 transgene) to delete IKK2 specifically in the haematopoietic cells of reconstituted mice.

Surprisingly our study revealed that loss of IKK2 in haematopoietic cells resulted in abnormally increased levels of IL-6 and severe gut inflammation necessitating euthanasia. IL-6, a pleiotropic pro-inflammatory cytokine produced and secreted by various cell types, including T cells, B cells, monocytes, macrophages and neutrophils^46,47^, functions in various biological processes, such as immune regulation, haematopoiesis, inflammation and oncogenesis^48^. Previous studies have shown that IL-6 is a direct target of the transcription factor NF-kB, since IL-6 expression is activated and regulated through the binding of NF-kB factors to regulatory sequences within the *IL-6* promotor^49–51^. This provides a link between IL-6 and IKK2. Hsu et al. stated that in their study, IKK2-deleted mice appeared to succumb to overwhelming generalised inflammation 6 months post poly(I:C)-mediated gene deletion^42^, i.e., considerably later than in our study. They hypothesised that this resulted from excessive IL-1 receptor signalling, causing neutrophilia^42^. In contrast, our study showed no changes in IL-1 beta cytokine levels, making it unlikely that the pathology observed was due to excess IL-1 receptor signalling. Instead, we found a significant increase in the levels of IL-6 in the sera of the IKK2-deleted animals. Histopathological analysis of major organs revealed that the severe destructive inflammation affecting the IKK2-deleted mice was manifested in the caecum and large intestine, with both tissues showing infiltration by inflammatory polymorphonuclear leucocytes and destruction of the epithelial architecture, both hallmarks of gut inflammation. Research in the field of gastroenterology has shown that pro-inflammatory cytokines, such as IL-6, have a fundamental role in controlling mucosal inflammation with imbalances between pro-inflammatory and anti-inflammatory cytokines known to hinder the resolution of inflammation, thereby leading to the perpetuation of inflammation and tissue destruction^52^. Cytokines, such as IL-6, have been shown to play a crucial role in the pathogenesis of inflammatory bowel diseases (IBDs) where they act as essential drivers of the inflammatory response^53^. It is, therefore, reasonable to assume that an imbalance between pro-inflammatory and anti-inflammatory cytokines caused by the abnormal increase in IL-6 could be the underlying cause of the severe intestinal inflammation we observed in mice whose haematopoietic cells had been deleted for IKK2. The results of our study and those from others have shown that deletion of IKK2 in haematopoietic cells causes severe neutrophilia due to a skewed lineage commitment. Therefore and because neutrophils, monocytes, T cells and macrophages all produce IL-6^47,52,54^, we hypothesise that an imbalance of cytokines caused by pathologically elevated levels of the pro-inflammatory cytokine IL-6 is most likely the underlying cause triggering intestinal inflammation in the IKK2-deleted mice. In support of this hypothesis, studies using mouse models of IBD have shown that blockade of IL-6/IL-6R signalling with monoclonal antibodies could suppress chronic intestinal inflammation^52^. Using such IL-6/IL-6R antagonists would allow future studies to test the hypothesis that abnormally increased IL-6/IL-6R signalling causes the gut inflammation in the IKK2- deleted mice.

Several independent groups have reported that a reduction of NF-κB activity by deletion of IKK2, NEMO, RELA(p65) or TRAF2/5 results in increased production of reactive oxygen species (ROS) and sustained c-JUN N-terminal kinase (JNK) activation in response to treatment with TNF^55–62^. This can culminate in cell death and, importantly, the prolonged JNK activation can be prevented through knockout of TNFR1. Pathological JNK activity has been implicated in a number of chronic inflammatory disorders, including colitis and IBD^63–67^, and this has been shown to regulate the synthesis of IL-6 and TNFα^68,69^. Moreover, Zhang et al.^35^ have shown that C/EBPβ expression, also implicated in *IL-6* gene transcription, is significantly increased in Lin^–^ cells from IKK2-knockout mice. Therefore, the elevated IL-6 levels we observed upon loss of IKK2 in the haematopoietic system may result from the activation of C/EBPβ and/or c-Jun/AP-1 in the absence of canonical IKK2-mediated NF-κB signalling.

Strikingly, mice containing 50% IKK2-deleted haematopoietic cells and 50% WT cells did not present with abnormally increased IL-6 serum levels, severe gut inflammation and the other phenotypes that resulted from the loss of IKK2 in all haematopoietic cells. This indicates that the loss of IKK2-mediated signalling in only half of the haematopoietic cells may not result in high enough TNF-induced ROS to reach the threshold to initiate a ROS/JNK-positive feedback loop that culminates in increased circulating IL-6, neutrophilia/thrombocytosis and destructive gut inflammation^41^. Alternatively, IKK2 deletion may sensitise myeloid cells to TLR-mediated killing, and it is possible that the presence of 50% haematopoietic cells in reconstituted mice allows the production of survival factors from these cells that limit the immunogenic cell death of the reconstituted IKK2-deficient cells, which would otherwise drive IL-6 production in other cell types (e.g., intestinal epithelial cells) and consequent inflammatory disease.

Notably, IL-6 has been shown to be a potent thrombopoietic factor stimulating megakaryocyte maturation (megakaryocytopoiesis) in the bone marrow, thereby promoting thrombocyte production leading to the release of platelets^70^. Thus, elevated IL-6 may explain the thrombocytosis observed in IKK2-deleted mice. Furthermore, IL-6 could contribute to T lymphocytosis in IKK2-deleted mice by expanding the lifespan of T lymphocytes^71–73^ that can themselves produce IL-6, thereby further elevating IL-6 serum levels and aggravating inflammation by establishing a positive feedback loop. This would be expected to cause an increase in T-cell numbers in IKK2-deleted mice despite an obvious lineage skewing towards myeloid commitment at the expanse of lymphoid progenitors, which appears responsible for the observed B lymphocyte and natural killer cell deficiency. It will be interesting to further define the precise mechanisms that underlie the increased IL-6 production and development of severe gut inflammation observed in mice whose haematopoietic cells have been deleted for IKK2.

## Materials and methods

### Haematopoietic reconstitution model and competitive haematopoietic reconstitution model

Mice harbouring floxed *Ikk1* or floxed *Ikk2* alleles were previously described^74,75^. These mice were bred with ROSA26 Cre-ERT2 mice (Jackson Laboratories) to generate C57BL/6 IKK1^(fl/fl)^; ROSA26 Cre-ERT2 or IKK2^(fl/fl)^; ROSA26 Cre-ERT2 mice. For transplantation experiments, IKK1^(fl/fl)^ or IKK2^(fl/fl)^ mice were crossed with IKK1^(fl/fl)^; ROSA26 Cre-ERT2 or IKK1^(fl/fl)^; ROSA26 Cre-ERT2 mice respectively to obtain C57BL/6 CD45.2^+^ E14 foetal liver cells homozygous for IKK1^(fl/fl)^ or IKK2^(fl/fl)^ and hemizygous for ROSA Cre-ERT2.

6–8-week-old female congenic C57BL/6 CD45.1^+^ recipient mice were lethally irradiated using two consecutive doses of 550 rad/5.5 Gy 2 h apart, with the second dose delivered 2 h prior to tail vein injection with 2 × 10^6^ CD45.2^+^ foetal liver cells harvested from donor wild-type, IKK1^(fl/fl)^; ROSA26 Cre-ERT2, or IKK1^(fl/fl)^; ROSA26 Cre-ERT2 E14 embryos as described above. The relative contribution to the bone marrow by donor CD45.2^+^ and recipient CD45.1^+^ cells was assessed in peripheral blood from a submandibular bleed 4 weeks post transplantation by flow cytometry using APC Cy7-anti-CD45.1 (A20) (BD Pharmingen) and FITC-anti-CD45.2 (104) monoclonal antibodies (BD Pharmingen). Blood from the same submandibular bleed was analysed to obtain a baseline blood profile of each mouse prior to gene deletion as a comparison. At 4 weeks after transplantation, tamoxifen (T5648, Sigma-Aldrich) was administered by oral gavage (200 mg/kg) on two consecutive days to delete the floxed target gene selectively in haematopoietic cells of the reconstituted mice in vivo. Successful deletion of the target gene was confirmed by Western blot analysis of bone marrow samples obtained from one sacrificed mouse of each genotype. Mice were closely monitored for any signs of illness post tamoxifen treatment, such as weight loss, anaemia, lethargy and irregular breathing, and culled if any of these signs were observed. At the point of sacrifice, blood, bone marrow (femurs, tibiae) and organs (sternum, thymus, lung, spleen, liver, kidneys, caecum and large intestine) were collected for analysis. Single-cell suspensions from bone marrow were obtained by extracting the bone marrow using a mortar and pestle to crush the bones and a 40-µm cell strainer. Red blood cells were lysed with NH_4_Cl buffer (156 mM NH_4_Cl, 11.9 mM NaHCO_3_ and 0.097 mM EDTA). Organs were prepared for histological analysis by fixation of the tissues in 10% buffered formalin, paraffin embedding, sectioning and subsequent staining with haematoxylin and eosin.

For complete blood count analysis, whole blood obtained from mice either by a submandibular bleed or cardiac bleed was analysed immediately using the Hemavet 950 system (Drew Scientific) for the haematopoietic reconstitution experiment or the Avida 2120 haematological analyser (Bayer) for the competitive reconstitution experiment. Some sample measurements were excluded from analysis due to technical errors.

For the competitive reconstitution experiment, 6–8-week-old female congenic C57BL/6 CD45.1^+^ recipient mice were lethally irradiated as described above, and transplanted with a total of 1 × 10^6^ wild-type, IKK1^(fl/fl)^; ROSA26 Cre-ERT2, or IKK1^(fl/fl)^; ROSA26 Cre-ERT2 CD45.2^+^ (donor 1) and 1 × 10^6^ CD45.1^+^/CD45.2^+^ heterozygous wild-type (donor 2) E14 foetal liver cells at a 1:1 ratio. Contributions of donor 1 (CD45.2^+^) and donor 2 (CD45.1^+^/CD45.2^+^) foetal liver-derived cells to peripheral blood cells in CD45.1^+^ recipient mice were analysed 4 weeks post transplantation by flow cytometry using staining with monoclonal antibodies against CD45.1 and CD45.2. The floxed target genes, *Ikk1* or *Ikk2*, respectively, were deleted in vivo by oral gavage with tamoxifen of mice 4 weeks post transplantation. Blood, bone marrow and organs were collected 3 weeks post gene deletion and analysed as described above.

Animals were randomly assigned to the experimental groups and transplanted with the respective foetal liver cells. The experimenters were not blinded and were aware of the genotype of the animals during experimentation. No statistical method was applied to predetermine the sample size of the experimental groups. The sample size was chosen based on relevant literature in the field.

Experiments with mice were conducted according to the guidelines and with the approval from the Walter and Eliza Hall Animal Ethics Committee (Parkville, Victoria, Australia) and the Alfred Medical Research and Education Precinct Animal Ethics Committee (Melbourne, Victoria, Australia). All experimental procedures were conducted in accordance with the Australian Code of Practice for the Care and Use of Animals for Scientific Purposes.

### Flow cytometric analysis

For the detection of cell surface markers, single-cell suspensions of bone marrow cells were washed with phosphate-buffered saline (PBS) supplemented with 1% heat-inactivated foetal bovine serum (HI-FBS) (A4503, Sigma-Aldrich) and incubated on ice with the following fluorochrome-conjugated monoclonal antibodies: PeCy7-anti-Sca-1 (D7), AF700-anti-CD45.2 (104), APC-anti-c-Kit (2B8), FITC-anti-CD41 (MWReg30), APC Cy7-anti-CD48 (HM48-1), PerCP Cy5.5-anti-CD16/32 (2.4G2), BV605-anti-CD3e (145-2C11), BV605-anti-B220 (RA3-6B2), BV605-anti-Gr-1 (RB6-8C5), BV605-anti-CD11b (M1/70), BV605-anti-Ter-119 (Ter-119), APC Cy7-anti-CD19 (1D3), APC-anti-Ter-119 (Ter-119), V450-anti-CD11b (M1/70), Biotin-anti-Nk1.1 (PK136), PeCy7-anti-Gr-1 (RB6-8C5), PE-anti-CD3e (145-2C11) (BD Biosciences), Pacific Blue-anti-CD105 (MJ7/18) and PE-anti-CD150 (TC15-12F12.2) (BioLegend). Streptavidin conjugated to FITC was used for the detection of biotin-conjugated antibodies.

Flow cytometric analysis was performed on the LSRIIW instrument (BD Biosciences, with BD FACS Diva software) and data were analysed using FlowJo analysis software (v10.1r7, FlowJo Enterprise). Dead cells were excluded from analysis by FSC/SSC exclusion, doublets by SSC-W/SSC-H and FSC-W/FSC-H exclusion and residual CD45.1^+^ recipient-derived cells by staining. Some samples were excluded from analysis due to technical errors.

### Western blot analysis

Single-cell suspensions from bone marrow were obtained by extracting the bone marrow from sacrificed mice using mortar and pestle to crush the bones and a 40-µm cell strainer to filter out debris. Total cell lysates were then prepared by lysing directly into SDS-PAGE gel loading buffer (250 mM Tris-HCl, 4% glycerol, 5% SDS and 0.25% bromophenol blue) at a cell density of 1.5 × 10^4^ cells/µL of buffer. Lysates were boiled for 5 min before proteins were resolved on 12% SDS-PAGE gels. Molecular weights were estimated using the commercially available MW markers Benchmark Pre-stained Protein Markers (Invitrogen). Proteins separated by SDS-PAGE were transferred onto PVDF membrane by electroblotting. PVDF membranes were blocked in 5% skim milk powder in TBS with 0.1% Tween-20, then probed overnight at 4 °C with primary antibodies against total IKK1 (Cell Signalling, Boston, MA, USA), total IKK2 (Cell Signalling, Boston, MA, USA) and β-Actin (Sigma-Aldrich, protein loading control). Bound primary antibodies were detected by the appropriate horseradish peroxidase-conjugated secondary antibodies (anti-Rabbit-IgG-HRP or anti-Mouse-IgG-HRP, both from Southern Biotech). Proteins were detected by chemiluminescence using an ECL kit (Super Signal West Dura Extended Duration Substrate; Thermo Fisher Scientific, Scoresby, Victoria, Australia) and developed using either film (GE Health Care Amersham Hyperfilm ECL; VWR international, Tingalpa, Queensland, Australia) or the ChemiDoc Touch System (Bio-Rad Laboratories).

### Measurement of cytokine levels in the serum of mice

Blood from mice was taken at the time of sacrifice by cardiac puncture, collected in an Eppendorf tube and left to clot overnight at 4 °C. On the next day, the serum supernatant was taken off and transferred into a fresh Eppendorf tube. To remove any remaining blood clots/blood cells, the serum was centrifuged at 3500 rpm for 10 min at RT. Pure serum was collected into a fresh Eppendorf tube and stored at −80 °C. The levels of cytokines were determined using ELISA (IL-1β from R&D systems, IL-6 and TNFα from eBioscience). For each set of samples, a standard curve was generated and sample concentrations were calculated using Prism, applying a nonlinear regression (curve fit) and the two-phase decay exponential equation. Some samples were excluded from analysis due to insufficient sample volume.

### Statistical analysis

Kaplan–Meier mouse survival curves were generated and analysed with GraphPad Prism Software Version 8 (GraphPad Software Inc., La Jolla, CA, USA). All data represent mean ± standard error of the mean, and statistical significance and *p* values were calculated using one-way ANOVA and Dunnett’s multiple comparison test unless otherwise specified. ^ns^*p* > 0.05, *p ≤ 0.05, **p ≤ 0.01, ***p ≤ 0.001 and ****p ≤ 0.0001. The variance was similar between statistically compared experimental groups. Mean, errors and *p* values were calculated using GraphPad Prism Software Version 8.

## Supplementary information

Supplementary Figure S1

Supplementary Figure S2

Supplementary Figure S3

Supplementary Figure S4

Supplementary Figure S5

Supplementary Figure S6

Supplementary Figure legends
