# Supplementary figures and images for: Deletion of IKK2 in haematopoietic cells of adult mice leads to elevated interleukin-6, neutrophilia and fatal gastrointestinal inflammation
Source: Cell Death Dis. 2021 Jan 4;12(1):28. doi: 10.1038/s41419-020-03298-9 (PMC7791118; doi:10.1038/s41419-020-03298-9)

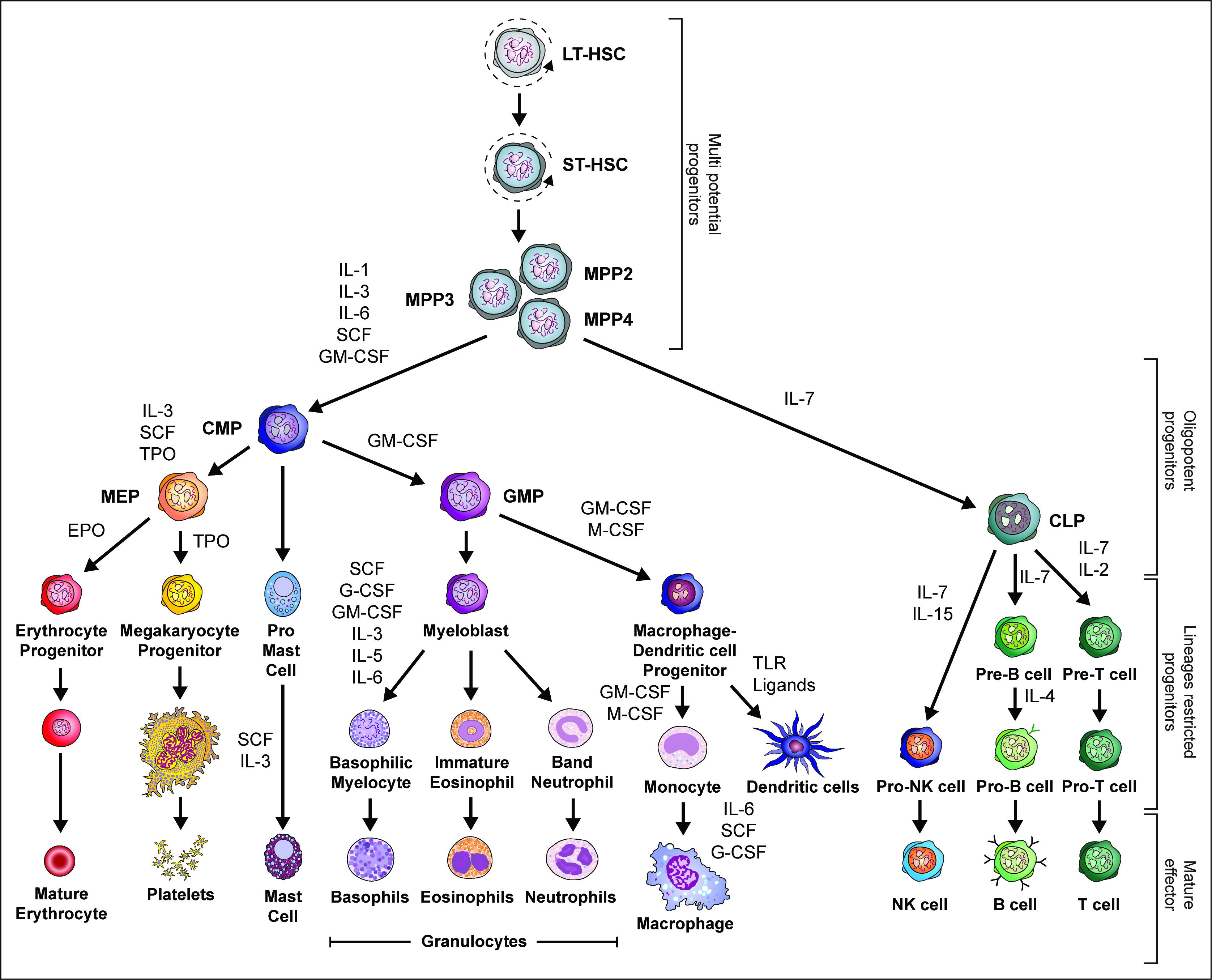

Supplement: Supplementary file 1 — Supplementary Figure S1 [file 41419_2020_3298_MOESM1_ESM.tif]

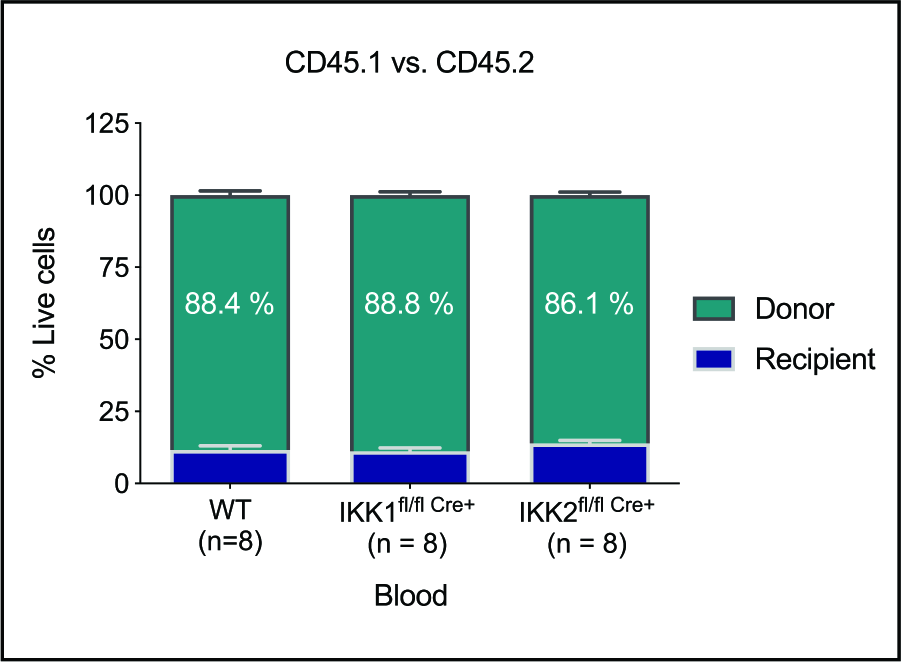

Supplement: Supplementary file 2 — Supplementary Figure S2 [file 41419_2020_3298_MOESM2_ESM.tif]

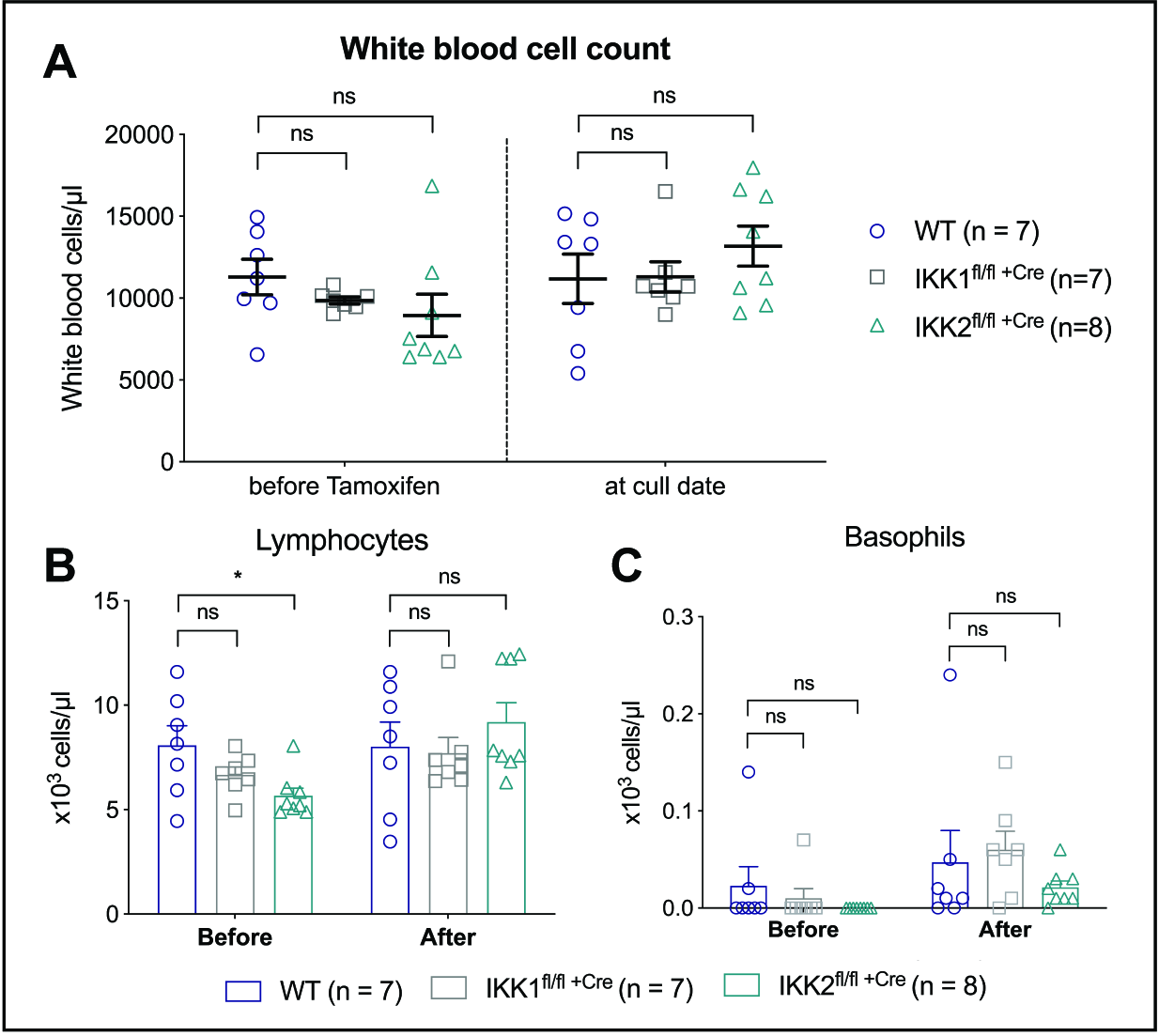

Supplement: Supplementary file 3 — Supplementary Figure S3 [file 41419_2020_3298_MOESM3_ESM.tif]

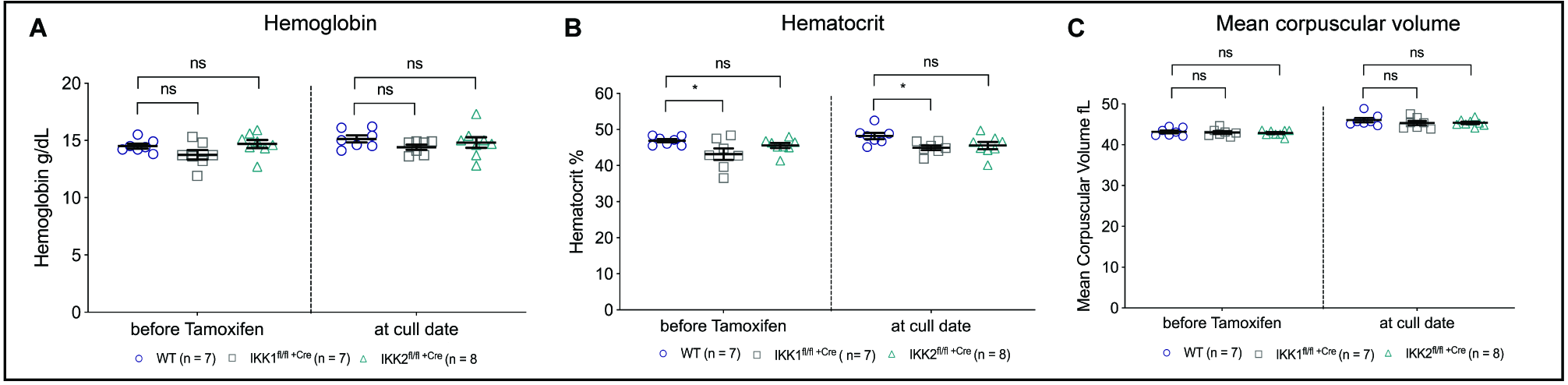

Supplement: Supplementary file 4 — Supplementary Figure S4 [file 41419_2020_3298_MOESM4_ESM.tif]

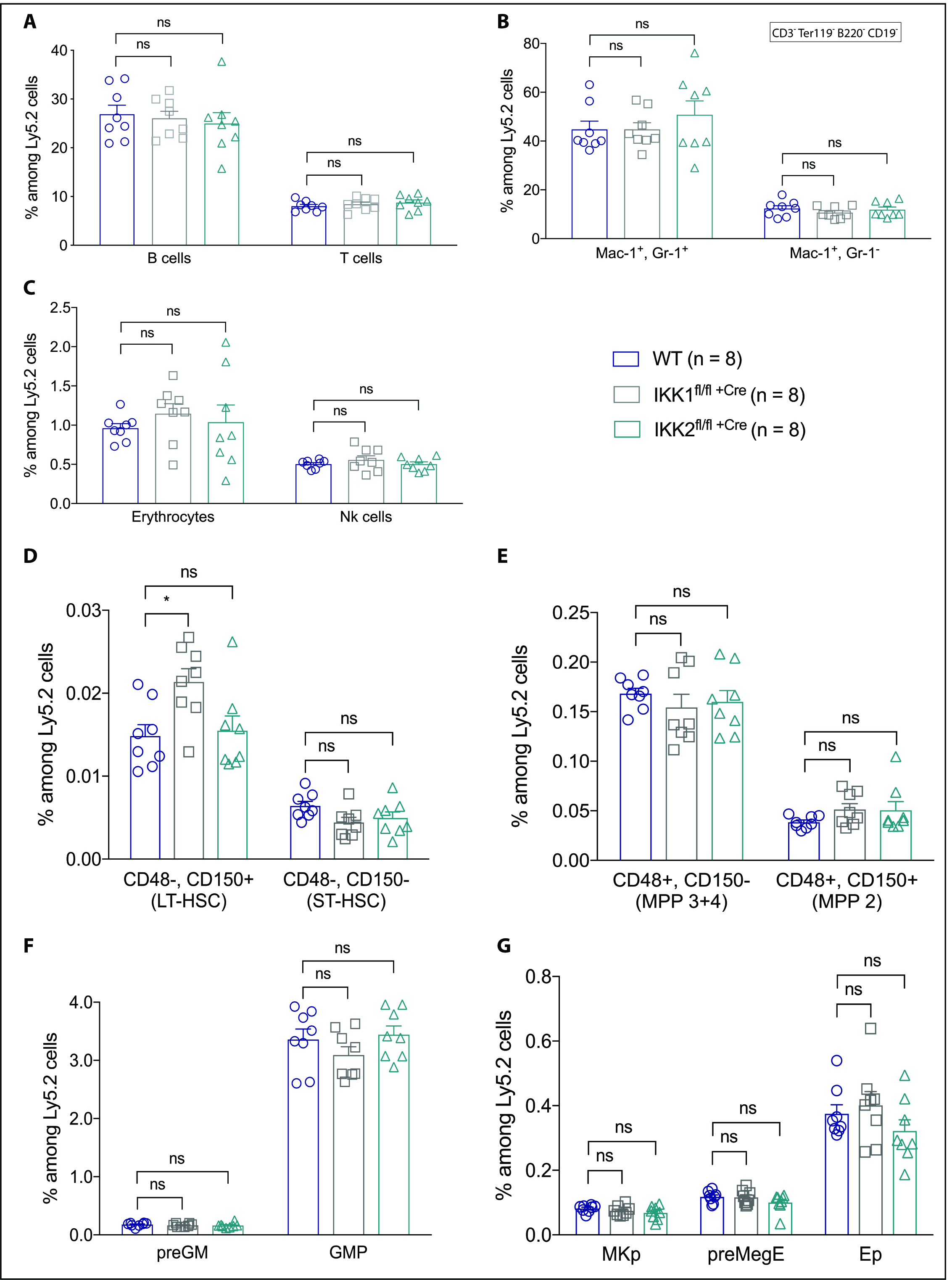

Supplement: Supplementary file 5 — Supplementary Figure S5 [file 41419_2020_3298_MOESM5_ESM.tif]

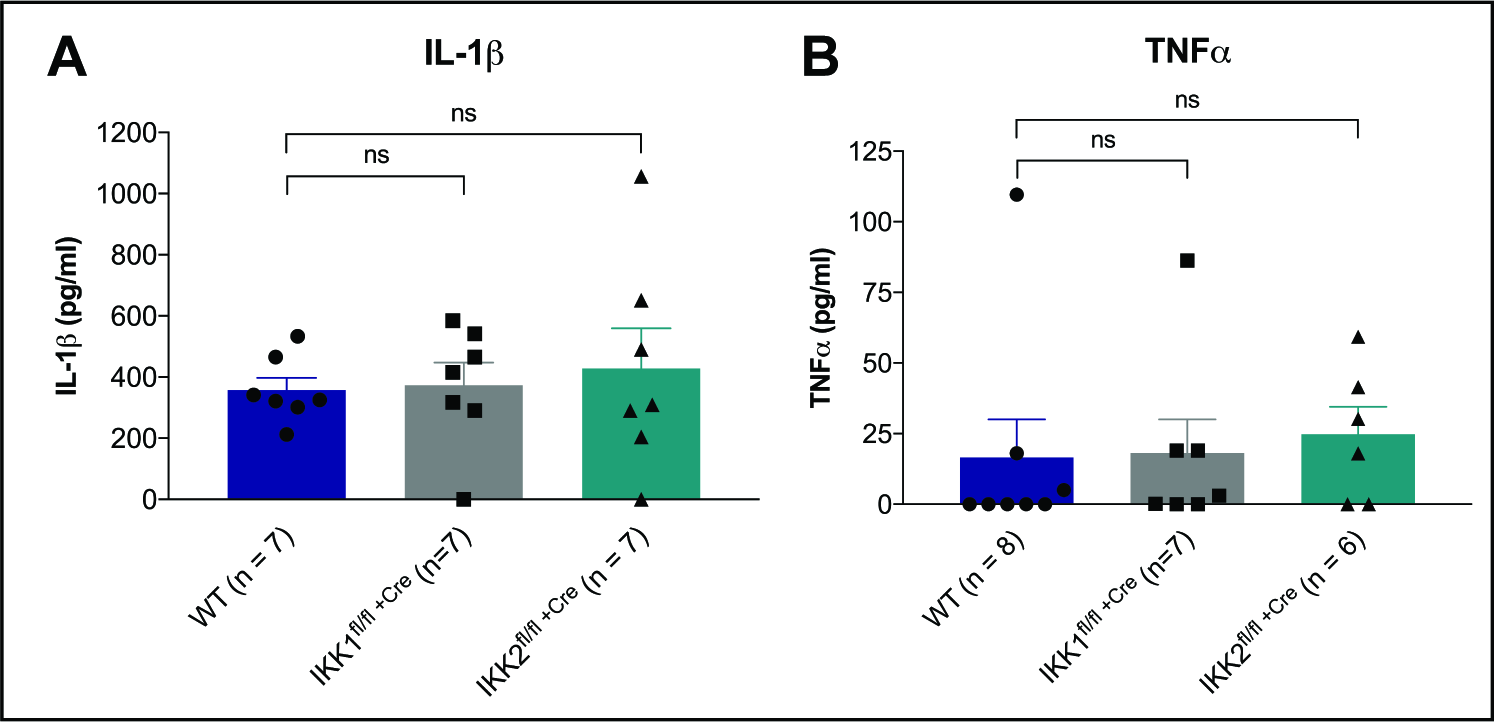

Supplement: Supplementary file 6 — Supplementary Figure S6 [file 41419_2020_3298_MOESM6_ESM.tif]
