# Supplementary material for: Deletion of IKK2 in haematopoietic cells of adult mice leads to elevated interleukin-6, neutrophilia and fatal gastrointestinal inflammation
Source: Cell Death Dis. 2021 Jan 4;12(1):28. doi: 10.1038/s41419-020-03298-9 (PMC7791118; doi:10.1038/s41419-020-03298-9)
Supplement: Supplementary file 7 — Supplementary Figure legends [file 41419_2020_3298_MOESM7_ESM.docx]

**Supplementary Figure S1: Haematopoiesis.**

Schematic of haematopoietic differentiation and maturation in vivo, displaying the hierarchical and stepwise progression of cells from haematopoietic stems cells (HSC) to more committed oligopotent myeloid and lymphoid progenitors, ultimately producing the various mature blood cell types of the myeloid and lymphoid lineages. Cytokines and growth factors essential during haematopoiesis are shown. LT-HSC, Long term HSC; ST-HSC, Short-term HSC; MPP, multipotent progenitor; CMP, common myeloid progenitor; CLP, common lymphoid progenitor; GMP, granulocyte-monocyte progenitor; MEP, megakaryocyte-erythroid progenitor; NK cell, Natural killer cell.

Supplementary Figure S2: Flow cytometric analysis of peripheral blood cells to determine the reconstitution efficiency in the competitive reconstitution assay.

Peripheral blood cells were stained for CD45.1 and CD45.2 cell surface markers and analysed by flow cytometry. Contribution of transplanted donor foetal liver cells (1:1 mix of CD45.2^+^ and CD45.1^+^/CD45.2^+^) to peripheral blood cells in recipient mice (CD45.1^+^) (mean + SEM; n = 8 for each genotype). Data are from one independent experiment.

Supplementary Figure S3: Analysis of peripheral blood profile in control and IKK deleted mice from the competitive reconstitution assay before gene deletion and at date of sacrifice.

(A) White blood cell count (WBC), (B) lymphocyte and (C) basophil count as assessed on the Hemavet blood analyser. Values for individual mice are shown and data are expressed as means ± SEM (A) or means + SEM (B + C) from 8 mice of each genotype of one independent experiment. ^ns^p>0.05 as determined by One-way ANOVA (WBC p = 0.2787; Lymphocytes p = 0.5171; Basophils p = 0.4265) and Dunnett’s multiple comparison test).

Supplementary Figure S4: Analysis of red blood cell parameters in control and IKK deleted mice from the competitive reconstitution assay.

(A) Haemoglobin concentration (HGB), (B) haematocrit (HCT) and (C) mean corpuscular volume (MCV) as assessed on the Hemavet blood analyser. Individual values, mean and SEM are shown of one independent experiment. ^ns^p>0.05 as determined by One-way ANOVA (HGB p = 0.3892; HCT p = 0.0403; MCV p = 0.4917) and Dunnett’s multiple comparison test.

Supplementary Figure S5: Flow cytometric analysis of the lineage distribution and the haematopoietic stem cell compartment of mice from the competitive reconstitution assay.

(A-G) Flow cytometric analysis of total bone marrow harvested from control and IKK1 or IKK2 deleted mice respectively at experimental endpoint. Percentages of (A) B-cells and T-cells, (B) myeloid populations Mac-1^+^/Gr-1^+^ and Mac-1^+^/Gr-1^-^, (C) nucleated erythrocytes and natural killer (Nk) cells, (D) CD48^-^ CD150^+^ long-term (LT) HSCs and CD48- CD150- short-term (ST) HSCs and (E) CD48^+^ CD150^-^ MPP 3+4 and CD48^+^ CD150^-^ MPP 2 cells, (F) pre-granulocyte-macrophage progenitors (pre-GM) and granulocyte macrophage progenitors (GMP) and (G) megakaryocyte progenitors (MKp), pre-megakaryocyte-erythroid progenitors (preMegE) and erythroid progenitors (Ep) among CD45.2^+^ cells in the bone marrow. Results are presented as means + SEM from 8 mice of each genotype of one independent experiment. ^ns^p>0.05 as determined by One-way ANOVA (Lin^-^ cKit^-^ p = 0.7341; B cells p = 0.7713; T cells p = 0.5251; Mac-1^+^ Gr-1^+^ p = 0.5080; Mac-1^+^ Gr-1^-^ p = 0.5264; Erythrocytes p = 0.6838; Nk cells p = 0.4838; LT-HSC p = 0.0156; ST-HSC p = 0.0927; MPP 3+4 p = 0.6527; MPP 2 p = 0.2798; preGM p = 0.6232; GMP p = 0.2833; MKp p = 0.2902; preMegE p = 0.2536; Ep p = 0.2939) and Dunnett’s multiple comparison test.

Supplementary Figure S6: Quantification of the inflammatory cytokines IL-1β and TNFα in sera of mice from the competitive reconstitution assay.

(A-B) ELISA analysis of the levels of IL-1β and TNFα in the sera of mice of the indicated genotypes at experimental endpoint. Data are shown as mean + SEM of one experiment, including individual values with n = 7-8 mice for control mice, n = 7 mice for IKK1 deleted mice and n = 6-7 mice for IKK2 deleted mice. ^ns^p>0.05 as determined by One-way ANOVA (IL-1β p = 0.8413; TNFα p = 0.8872) and Dunnett’s multiple comparison test.
